# 7-Steps to Creating an Effective Simulation Experience for Educators in the Health Professions: an updated practical guide to designing your own successful simulation

**DOI:** 10.15694/mep.2019.000166.1

**Published:** 2019-09-10

**Authors:** Donna Krawczyk

**Affiliations:** 1 Michener Institute

**Keywords:** curriculum design, learning outcomes, critical reflection

## Abstract

This article was migrated. The article was marked as recommended.

Creating a simulation experience for learners can be a daunting task for educators. Through a literature search, this guide outlines a feasible method to effectively execute a successful learning experience for future health professionals through creating your own simulation event from scratch. By organizing this learning strategy into steps, an educator can easily reproduce their very own simulation and offer a highly recommended tool for enhancing health professional education within their in-class or e-learning curriculum. Reaching your students through simulation as a learning strategy does not have to be expensive nor does it have to be a complete re-enactment. To offer a simple but purposeful, clinically relevant simulation is also well remembered for real-life use. Simulation provides a framework for an experience to happen where a student is to engage prior knowledge into practice and the educator takes a facilitative role (Levine *et al*., 2003). Knowing when and where to use simulation and understanding its effectiveness is key in reaching your learners as well as offering appropriate debriefing. This paper will outline the skills you need and support your choices in which simulation event best suits the required tested outcome.

## Objective

This innovative 7-step process to designing your own successful simulation follows 3 main learning objectives for the educator. Firstly, recognizing the value and relevance of the concept of simulation for students in the health profession is important. Secondly, we hope this paper will help you design your own simulation event from scratch and learn the ability to give effective feedback through facilitated debriefing. Thirdly, by understanding when, where, how and being creative you will be ready as an educator to apply this learning strategy to you students and incorporate it appropriately in your curriculum ( Irby and Wilkerson, 2003).

## Introduction

The potential applications of simulation are far-reaching ( Ziv, Ben-David and Ziv, 2005). Simulation is in its roots, based on an adult educational theory that stems from Dewey’s experiential learning theory (Levine *et al*., 2013; Brubacher *et al*., 2016). It encourages the student to gather more information through experience from what they already know or learned in class (Levine *et al*., 2013). Now that we know simulation is often used as an instructional design in the health professions we, as educators, must know where and how to fit it into a curriculum to maximize effectiveness. Please be sure to consider your class time frame and understanding your “zone of simulation” ( Chiniara *et al*., 2013). Asking yourself: Do you have enough time to accommodate a simulation experience? It could take 2-3 days of your schedule to be effective for students to grasp and find practical use of this type of instruction ( Chiniara *et al*., 2013). Simulation has been an integral part of health professional education all over the world. In the context and principles of health profession education, the use of simulation has been proven an effective learning strategy (Levine *et al*., 2013). The unpredictable clinical setting of any medical profession in real-life gives simulated experiences an invaluable teaching opportunity. Healthcare has been improved through students in the health professions to learn from properly executed simulation, that includes a constructive debriefing, because it leads to fewer medical errors and a better quality of care ( Irby and Wilkerson, 2003). In this guide we instruct clinical educators on how to effectively design your own successful simulation. By using this 7-step process, teaching a medical event through simulation will be more interesting for students, as we have seen. Simulation is known as an engaging technique that replicates clinical scenarios for applied learning in the health professions ( Ziv, Ben-David and Ziv, 2005). Here, we take the sometimes complicated concept to execute and create an easy to follow procedure that will make the educators simulation experience relevant, evidence-based and exciting for students. Although simulation can be useful as a “see one, do one” concept of learning, it is most useful to the student in learning situations that need high accuracy and where opportunity to encounter this situation is not common in the medical field ( Chiniara *et al*., 2013). For example, a mass casualty emergency. A health professional must have access to continually learn through repetition of the simulation in order to deal with uncommon occurrences in order to have the most impact on saving lives ( Chiniara *et al*., 2013). Keep in mind, simulation is interactive, not exposing the patient to risk and an important part of educating the learner because it reproduces an aspect of medicine that may not be encountered during training but ultimately important to know (Levine *et al*., 2013).

## Step-by-step methodology

As you read, you will recognize the value and relevance of the concept of simulation through the following steps:

### Step 1. Finding the target learning group

Is it surgeons in the operating room or a collaboration of students learning about teamwork communication? Could it also be a single learner who needs to practice a specific skill on a part of the body? For this step you ask yourself, who are the students you are teaching in order to move on to step 2. The role of simulation in educating novice health professionals extends from refining psychomotor skills to teaching interprofessional care, nontechnical skills that may be encountered in real life and learning to deal with critical care situations (Levine *et al.*, 2013). Going back to the “zone of simulation”, it is studied that it is most advantageous when using simulation as a learning strategies to pose to your target group situations that complement learning most in occurrences where the greatest potential for a negative impact to the patient is present ( Chiniara *et al*., 2013). This is not to say that simulation experiences from the other end of the spectrum of common occurrences is not appropriate, however, these situations are less effective when using simulation as a learning strategy and perhaps another strategy may be more useful ( Chiniara *et al*., 2013).

### Step 2. Finding a simulation location

Finding a place on campus or an off-campus location simulation may be out of your control. It could be enough space for a computer or you may be lucky enough to have access to a simulated room. Having a place to conduct the simulation is important because it is key for the students to have access to the simulation after hours and use it to practice their skills in order to be ready for real-life situations.

### Step 3. Simulation purpose

Figuring out the main learning objective of your simulation gives the simulation a purpose and so competencies can be tested ( Chiniara *et al*., 2013; Levine *et al*., 2013). Some commonly tested competencies where students have hands-on experience and interact in simulation are: knowledge; techniques and procedures; history, examination and counseling; clinical reasoning and patient management; teamwork and crisis management; and finally, ethics and beliefs.

### Step 4. Simulation delivery

Once you figure out the purpose of the simulation based on the students outcomes needed, the simulation delivery can be matched. Simulation can be applied in 4 different ways: computer based, simulated patient, simulated clinical immersion and procedural simulation ( Chiniara *et al*., 2013).

Computer based simulation is considered a virtual reality setting for the student. In this case of simulation, the student is commonly watching a screen where a scene unfolds. For example, it could involve dealing with an interprofessional conflict like a difficult phone call with a colleague ( Maran and Glavin, 2003). A simulated patient could be a payed actor, an actual patient volunteer or another student where there could be an uncommon sign or symptom. In a clinical immersion simulation, reconstructing the environment becomes important as it is an exact re-enactment ( Chiniara *et al*., 2013; Maran and Glavin, 2003). For example, a simulated operating room involving a complication during surgery ( Brubacher *et al*., 2016). Lastly, a procedural simulation has the purpose of enhancing a motor skill through repetition like the practice of draining an abscess in order to refine a skill ( Chiniara *et al*., 2013; Brubacher *et al*., 2016; Augenstein, Yoshida and Solari 2016). Using table 1 below, educators can learn how to match step 3 to step 4 to optimize the simulation style and purpose through a thoughtful outlined combination.

**Table 1.** Highlights a method to easily understand when to use a specific type of simulation in order to optimize the learning strategy purpose.

Step 3: Simulation Purpose	Step 4: Simulation Delivery
Ethics and beliefs; knowledge	Computer-based
History, examination, and counseling; knowledge	Simulated patient
Teamwork; crisis management; knowledge	Simulated clinical immersion
Techniques and procedures	Simulated patient

### Step 5. Collecting resources

In Step 5, asking yourself what other resources the simulation will need. By collecting more resources, teaching the simulation can be more effective. This depends on what you are teaching and its level of technique. Thinking about what materials you may use for your students to learn the simulation purpose will be based on financial limitations. There are two choices to consider: low fidelity versus high fidelity ( Brubacher *et al*., 2016; Augenstein *et al*., 2016). In demonstrating the difference between low fidelity versus high fidelity it is seen that high fidelity is much more involved and expensive while low fidelity options could be simply a pen and paper or something home made. The low fidelity simulation may not be as real but financial restraints could be a factor in your choice of what resources you will need to conduct your simulation.

### Step 6. Determining the style of simulation

Step 6 helps the educator decide on when to choose between two evidence-based simulation exercises that would be ideal for their previously considered Steps 1 to 5. In Step 6, the simulation will be what is known as “student-centered” or “facilitator-centered” ( Ker and Paul, 2010; Maran and Glavin, 2003). The criteria for choosing a facilitator-centered simulation means the teacher is the focus of the step-by-step learning simulation. Here, the facilitator is in control, the student knows the outcome of the simulation because it is something learned previously in class and the simulation offers a space to practice. In this case, you use Ker’s STEPS mnemonic. So, Ker’s STEPS mneominc is used as an enhanced “see one, do one” where key steps are in place to follow for the student to benefit fully from a procedural technique that is meant to be practice ( Ker and Paul, 2010).

In the first step, the “S” stands for setting a foundation ( Ker and Paul, 2010). The facilitator’s duty is to offer an overview to a group of learners where the ultimate goal is known by the students because it was taught in class or online prior to the simulation ( Ker and Paul, 2010). The “T”, stands for tutor in real time where the facilitator must make sure all students can clearly observe the simulation ( Ker and Paul, 2010). Then, “E” is when the procedure is repeated with an explanation ( Ker and Paul, 2010). “P” allows the students to practice, one-by-one with supervision by the facilitator and peers to offer feedback during the simulation as they learn it once ( Ker and Paul, 2010). The final “S” of STEPS is to encourage subsequent practice and allow the learning to continue after hours with other students ( Ker and Paul, 2010).

The criteria for choosing a student-centered simulation is opposite to Ker’s STEPS mnemonic as it focuses on the student being in control of the simulation ( Alinier, 2011). The student is forced to use critical thinking to find the simulation purpose and in the facilitator is somewhat in the background, perhaps provided some simple hints if the student is completely on the wrong track ( Alinier, 2011). In this case, you use Alinier’s scenario-based learning. In Alinier’s scenario-based learning, a scenario is in place where the facilitator gives an introductory statements of information but then there is an objective for the student to figure out on their own. The scenario in Alinier’s simulation offers a situation where the student has to decide on their own and is relateable to future clinical experiences ( Alinier, 2011).

The student is received at an ambiguous simulation, where it is scenario-based learning ( Alinier, 2011) Unlike, Ker’s STEPS, Alinier’s version often offers information that is conflicting because the outcome is unknown to the student ( Alinier, 2011). The student takes control of the simulation and the facilitator observes, as the event unfolds, all staff or faculty involved are able to use codes or communicate through devices so to guide the student in the right direction because the goal is not known ( Alinier, 2011). For example, the student recognizes a crisis situation and offers to help based on what was learned in class in order to surmise the objective of the simulation that could be prompted by facilitators involved in the simulation as the student continues through it.

### Step 7. Effective Debriefing

The most important part of an effective simulation experience is the debriefing after the simulation has been completed by the students ( Rudolph *et al*., 2007) The only way to really reach the student through simulation is to have a supportive, reflective and well-facilitated debriefing. It is easy for the educator to consider debriefing like feedback or evaluation but it is truly considered a process of facilitated self-reflection for the student to review the concrete experience she has just actively learned and can learn again based on self-reflection facilitated through debriefing ( Rudoplh *et al.*, 2007; Ker and Paul, 2010). The goals of debriefing are to allow the learner to explain, analyze and synthesize information and emotional states experienced during the simulation in order to improve performance in similar situation in the future ( Rudolph *et al*., 2007). The educator becomes a facilitator and observes the student throughout the simulation and through gathering information from what they see with what the student expresses post-simulation, an effective debriefing can be accomplished. Using direct observations as examples for the student to reflect on is key. Through descriptive and detailed input, the facilitator can direct the student’s learning through the simulation. Remember, the facilitator is best not to use evaluative discussion but alternatively propose encouraging language that are specifically in context to the simulation just conducted ( Rudolph *et al.*, 2007). It is important not to generalize information but to be prepared to offer debriefing that can limit behaviours that are remediable ( Rudolph *et al*., 2007). By focusing on the activities, a debriefing experience becomes respectful and trusting and what is expected from the simulation can be corrected by the student and used professionally ( Rudolph *et al*., 2007).

The debriefing is a much longer experience than the introduction of the simulation (which simply orientates the student) and the actual simulation itself. For example, be prepared to offer at least 45 minutes of facilitated debriefing post-simulation, that could have only been 15 minutes. The debriefing has to be immediately after the simulation and by taking notes based on the objective of the simulation, a debriefing can also involve a video because it must include direct observations in order to promote improvements in performance. The reflective practice method was developed by Rudolph *et al*., (2007). This includes helping the student see their actions in the simulation as a frame of mind that develops into actions and results in the outcome ( Rudolph *et al*., 2007) The point of the debriefing is to aid the student in finding their frame of mind during an activity in the simulation that resulted in the successful or unsuccessful result ( Rudolph *et al*., 2007). By understanding what the student was thinking about, the facilitator can help the student understand the output and through debriefing may have the opportunity to change the action to develop an expected result in future work ( Rudolph *et al*., 2007).

The reflective practice method encorporates the Schon Theory of Reflection where an action, either good or bad, is based on the student’s thought processes prior to conducting the action ( Schon, 1983). By facilitating a student to understand their thought processes, the student can use this method in developing proper professional practice ( Eppich and Cheng, 2015; Schon, 1983). The central idea that has created an action in a student in the stimulation is the key defining characteristic to discuss and allow reflection in order to help modify the thought process or frame that lead to a, perhaps, wrong action ( Ker and Paul, 2010; Rudolph *et al*., 2007; Schon, 1983). As an educator, debriefing offers a time for the learner to look at their own reactions and understand their own thought processes that occurred during the simulation ( Rudolph *et al*., 2007). The debriefing is a crucial learning moment where the facilitator takes the opportunity to resolve actions that are observed during the simulation that may have resulted in poorer outcomes than would be expected ( Rudolph *et al*., 2007). A technique known as critical reflection allows the role of adult education and simulation to intersect ( Sinz *et al*., 2014). Critical reflection helps the learner change their frame of thinking and result in corrected actions. For example, encouraging the student to figure out their own mislead thinking to an inappropriate action in a simulation is done with reflective listening and inquisitive prompting with questions. As this conservation occurs in a safe environment, critical reflection allows the learner to understand their need to change an action so as to apply it in their future career and succeed in real situations ( Schon, 1983; Sinz *et al*., 2014). When the facilitator sees the learner as thinking in frames, new frames can be understood in order to promote a change or shape an action that will achieve the optimal outcome - in turn, this newly acquired understanding in a situation in real life can help frame decision making that will minimize errors and enhance patient safety ( Rudolph *et al*., 2007). It has been shown that during a simulation, as is in clinic, the actions stem from rational decisions, which could unintentionally result in an error in care ( Rudolph *et al*., 2007). So, it is the role of the facilitator to find these intentional but ill actions, prompt the leaner to realize the consequence of their thinking and surface them so that new results may be formed ( Rudolph *et al*., 2007). This process makes simulation learner-centered, self-reflective and offers a chance to collaborative ask questions that create insights choices that develop positive actions ( Eppich and Cheng, 2015 and Schon, 1983). In the following example, a dialogue between a student and facilitator exhibits the critical reflection technique. Here, the learner does not initially understand that the scenario of resuscitating a patient was meant to end in death because the true objective of the simulation is to learn to deal with the death of a patient and offer information to the simulated family member afterwards.

“So, how did you feel during that simulation?”

“My resuscitation was not successful and I was confused about what I should do next.”

“It sounds like the simulation was stressful for you.”

“Yes, I feel like I did everything correctly but the outcome was not expected.” “When looking at your other options, it looks like you did not have much.”

“Actually, yes, my reasoning and the use of the equipment was correct to me. After seeing my techniques fail, I realized I had no other choice but to deal with the results of my actions.”

“That clarifies the objectives because how do you deal with death? The purpose of the simulation seemed predictable at first and I understand your reasoning because the unpredictable nature of this simulation was the primary objective.”

A good way to let the learner know you understand their confusion or questions during a simulation is by simple paraphrasing their thoughts during the simulation and restating the comments in a question form that focuses on the simulation purpose. This reflective listening gives the power of critical reflection to the leaner to help them clarify their decisions and a method to handle the situation or solve the problem on their own in the next similar situation. This technique allows non-judgmental facilitating and acceptance of the learner’s dilemma ( Rudolph *et al.*, 2007 and Alinier, 2011). So, through debriefing, the student is directed at targeting the points of improvement by allowing their frame of mind to gather and build on the simulation to improve future patient care. The objective of the simulation is made clear and future use of the simulation can develop a reflective practitioner by noticing and asking questions by the facilitator to find out the student’s reasoning into an action ( Rudolph *et al*., 2007; Eppich and Cheng, 2014; Schon, 1983). Using inquiry based on observed actions that need improvement during the simulation, the student is debriefed in an effective way that can develop into accuracy and extracting thoughtful practice ( Rudolph *et al.*, 2007 and Sinz *et al*., 2014).

## Conclusions

Simulation is an instructional strategy that is used in addition to course work and fits into a curriculum for health professionals to improve on a previously learned concept. Through this 7-step process, an educator can create their very own simulation experience that is evidence-based and helps the learner reach curriculum objectives. The student is prepared in class for the information meant to be gathered in the simulation and objectives are reviewed and gathered post-simulation using facilitated debriefing methodology. Watching videos on this topic for educators to improve their facilitating skills is encouraged to improve and relay concrete and descriptive input and questioning that can provide a thoughtfully engaging debriefing session.

## Take Home Messages

• Simulation is an educational tool that offers a controlled environment to improve technique and is important in minimizing practitioner errors through practice
• By using this 7-step process, the sometimes overwhelming instructional design of simulation can be simplified and conducted effectively
• The educator takes on the role of a facilitator for the learner to engage prior knowledge into action
• Reaching the learner’s thought process during a simulation is necessary to understand to effectively offer successful debriefing post-simulation and encouraging self-reflection is key to reaching the expected simulation objective

## Notes On Contributors

During her studies at The University of Toronto, Donna attained her certification in clinical teaching and did a workshop in simulation. Currently a clinical adjunct professor in chiropody at the Michener Institute, Donna is working as a chiropodist and passionate about teaching in the health professions.
