# Supplementary material for: 7-Steps to Creating an Effective Simulation Experience for Educators in the Health Professions: an updated practical guide to designing your own successful simulation
Source: MedEdPublish (2016). 2019 Sep 10;8:166. [Version 1] doi: 10.15694/mep.2019.000166.1 (PMC10712514; doi:10.15694/mep.2019.000166.1)
Supplement: Peer review history

# Reviewer report: Reviewer response for version 1

**DOI:** 10.21956/mep.19880.r30065

**Published:** 2019-09-26

**Authors:** Suzanne Lady

**Affiliations:** 1 University of Western States

This review has been migrated. The reviewer awarded 4 stars out of 5 This well-done paper guides the educator in creating successful simulations with students. The authors give examples of what specific simulations might look like and walks the reader through a 7-step process that enables the educator to begin planning simulation experiences. I found the last section on debriefs most effective and meaningful. The debrief is the time that learning occurs and if done well can have lasting effects. If this is done poorly this becomes a point of frustration for the learner. The authors describe a successful debrief session giving examples of dialog between student and facilitator and takes the reader through the components necessary for successful active learning. This is an important paper for anyone interested in simulation and having more meaningful active learning experiences for learners.

Reviewer Expertise:

NA

No decision status is available

# Reviewer report: Reviewer response for version 1

**DOI:** 10.21956/mep.19880.r30066

**Published:** 2019-09-11

**Authors:** Gert Van Zyl

**Affiliations:** 1 University of the Free State

This review has been migrated. The reviewer awarded 4 stars out of 5 A very well written article on a very important topic. The 7 Step approach will help educators (more so for those teaching in simulation for the first time)to plan an engagement properly. The majority of the information relating to simulation is not new, but presented in a well structured format. A contribution to the area of simulation.

Reviewer Expertise:

NA

No decision status is available
